# Girls, Don’t Marry in a Hurry

**Published:** 1892-06

**Authors:** 


### GIRLS, DON'T MARRY IN A HURRY.

Young girls, wait until you are at least 25 before you think of marrying. All tastes change between the ages of 16 and 30. The books you read, the games you enjoy, the milliner's skill you trust to, the friendships you cultivate, all are changing; why should not the tastes and fancies of the soul? The age that feeds upon Mrs. Southworth and Mrs. Holmes is vastly unlike the age that demands stronger mental food. The hero you are ready to worship at 18 will not be a hero to you, ten chances to one, at 28.

Wait until your tastes settle and the possibilities within you have found their level before you fix upon an unalterable destiny. For I tell you, whether it brings sorrow or joy, the choice you make is an irrevocable one. The moon may go back and be a crescent ere the first quarter is reached, the rose re-enfold itself within the calyx of the bud, or the sultry noon renew the freshness of the dawn before the circle of the day is spanned, with greater ease than a woman, can resume the lightness of her maiden fancies after the die of her wedded lot is cast.

